# Caffeine exacerbates exercise‐induced gut cell damage and is influenced by ADORA2A genotype but not CYP1A2 genotype: A preliminary study

**DOI:** 10.14814/phy2.70673

**Published:** 2025-11-19

**Authors:** Glen Davison, Alexander T. Carswell, Pauline Baron, Borja Martinez‐Gonzalez

**Affiliations:** ^1^ School of Natural Sciences (Sport, Exercise, and Rehabilitation Sciences) University of Kent Canterbury UK; ^2^ Norwich Medical School, Faculty of Medicine and Health Sciences University of East Anglia Norwich UK; ^3^ Unité de Recherche Pluridisciplinaire Sport Santé Société Université du Littoral Côte d'Opale Dunkerque France; ^4^ Kent and Medway Medical School University of Kent Canterbury UK

**Keywords:** caffeine sensitivity, cycling, gastrointestinal, genetics, polymorphism

## Abstract

Endurance exercise may be associated with acute damage to intestinal epithelial cells. The effect of caffeine supplementation, and whether this is influenced by common genetic polymorphisms (ADORA2A: rs5751876 and CYP1A2: rs762551), is not currently known. Participants (*n* = 18 men and women) ingested caffeine (3 mg/kg body mass) or placebo 45 min before cycling (20 min at 70% maximal oxygen uptake followed by a 15‐min performance time‐trial). Plasma intestinal fatty acid binding protein (iFABP) was measured pre‐supplementation, pre‐ and post‐exercise. Four‐way mixed ANOVAs revealed significant main effects of treatment, time, and treatment × time interaction (*p* < 0.05). Post hoc tests revealed a post‐exercise increase in plasma [iFABP], which was greater with caffeine; and a trial × ADORA2A genotype interaction *p* = 0.021, with further post hoc analysis revealing a significant post‐exercise increase only in ADORA2A TT (“high sensitivity”) participants in the caffeine trial (increase ~109%, *p* = 0.027, vs. 48% increase *p* > 0.05 for “low sensitivity” participants). There were no other main effects or interactions (all *p* > 0.05). Acute damage to gut cells caused by endurance exercise may be exacerbated by caffeine, especially in sensitive individuals. The potential implications of this for gastrointestinal responses to exercise warrant further examination.

## 1 INTRODUCTION

Endurance exercise may be associated with acute damage to intestinal epithelial cells (gut cells). Caffeine supplementation is a common practice in endurance athletes, due to the well‐established ergogenic effects. However, there is also potential for caffeine supplementation to have undesirable side effects on the gut, some of which have the potential to counteract the performance‐enhancing effects (de Souza et al., 2022; Pallarés et al., 2013; Wilson, 2016).

Snipe et al. (2017) and Houghton et al. (2023) have demonstrated that acute carbohydrate (CHO) intake before and during exercise provides protective effects against exercise‐induced perturbations to gastrointestinal permeability and cell damage markers. However, as demonstrated by Van Nieuwenhoven et al. (2000), the addition of caffeine to a CHO‐containing beverage negated the beneficial effects of the CHO beverage. For example, Van Nieuwenhoven et al. (2000) reported an increased post‐exercise lactulose‐to‐rhamnose ratio (indicating greater increases in gastrointestinal permeability) when caffeine was added to the CHO beverage compared to the same CHO beverage without caffeine. Further support for this was provided by Wilson (2016) who conducted a field‐based study in triathletes and reported a positive relationship between caffeine intake on the morning of a race and the incidence of lower gastrointestinal symptoms during the run phase. Pallarés et al. (2013) also reported some associations between caffeine intake and gastrointestinal symptoms and discomfort following resistance and maximal sprint cycling exercise. Indeed, a recent systematic review identified gastrointestinal problems in 32% of cases immediately post‐exercise, and 72% after 24 h, for caffeine doses >6.1 mg/kg body mass (de Souza et al., 2022).

There are multiple mechanisms via which caffeine may affect the gastrointestinal system and/or contribute to gastrointestinal symptoms or side effects in some individuals. Caffeine has been suggested to inhibit glycoprotein secretion from mucous‐producing cells (Hamada et al., 1997), which may have a negative effect on protective barrier properties and functions at epithelial/mucosal surfaces. Caffeine may also stimulate colonic motility, and gastrointestinal fluid secretion, which could cause mechanical stress, contributing to symptoms and resulting in further damage to the barrier and epithelial cells in the gastrointestinal tract (e.g., Wald et al., 1976). Caffeine is also known to enhance sympathetic nervous system activation and increase catecholamine responses to exercise (Graham & Spriet, 1995) further augmenting the exercise‐induced redistribution of blood flow (hypoperfusion) from the splanchnic region (to exercising muscles), which can contribute to exercise‐induced disturbance to gastrointestinal barrier function (van Wijck et al., 2013).

It has been noted that there is large inter‐individual variability in how people respond to caffeine in general (Grgic et al., 2020), with genetic factors possibly contributing to this variability (e.g. Pickering & Kiely, 2018; Wilson, 2022). For example, the rs5751876 single‐nucleotide polymorphism (SNP) in the ADORA2A gene (encoding the adenosine A2A receptor) has been used to categorize individuals as having “high” (TT genotype) or “low” (CT or CC genotype) caffeine sensitivity; and the rs762551 SNP in the CYP1A2 gene (encoding the P450 enzyme) has been used to categorize individuals as “fast” (AA genotype) or “slow” (AC or CC genotype) caffeine metabolisers (Nehlig, 2018). However, the theory that genetic factors contribute to inter‐individual variability in gastrointestinal responses to caffeine (and exercise) has not yet been examined experimentally. Therefore, the aim of this study was to determine the effects of acute caffeine ingestion on a marker of gut cell damage and whether this is influenced by commonly occurring SNPs in ADORA2A (rs5751876) and CYP1A2 (rs762551) genes.

## 2 METHODS

This exploratory study was conducted using samples from our previously published study on the influence of these SNPs on caffeine's effects on physical and cognitive performance (Carswell et al., 2020) and was approved by The University of Kent, School of Sport and Exercise Sciences Research Ethics and Advisory Group (ref: Prop 106_2017_18 and amendment ref: Prop 106_2017_18), with all procedures conducted in accordance with the Declaration of Helsinki. Eighteen young, healthy men (*n* = 12) and women (*n* = 6) (age 23.6 ± 4.1 years; height 175 ± 9 cm; body mass 70.7 ± 9.9 kg; VO_2_max 47.4 ± 9.0 mL/kg/min) volunteered to participate in this double‐blind, randomized crossover study and provided written informed consent. All participants were without any known immune, cardiovascular or metabolic disease; were free from injury or illness; and were not taking any medications. Further information on physiological responses to exercise, exercise, and cognitive performance have already been reported in Carswell et al. (2020).

### 2.1 Preliminary measures and familiarization

Maximal oxygen consumption (V̇O_2max_) was determined using a step incremental exercise test on an electromechanically braked cycle ergometer (Excalibur Sport, Lode, Groningen, The Netherlands). The test began at a power output of 100 W and increased by 25 W every 3 min up to 200 W, after which the power output increased by 25 W every minute until volitional exhaustion. Pulmonary gas exchange was measured breath‐by‐breath throughout the test (Metalyser 3B, Cortex Biophysik, Leipzig, Germany). After 30 min rest, participants were familiarized with the experimental procedures. Participants cycled for 20 min at the power output estimated to elicit 70% V̇O_2max_. After 5 min rest, participants were instructed to perform as much work as possible during a 15 min cycling time trial, as described in Carswell et al. (2020) using the methods of Jeukendrup et al. (1997).

### 2.2 Experimental procedures

On two occasions after an overnight fast, participants reported to the laboratory at the same time of day, having avoided consuming caffeine for 48 h and strenuous exercise for 24 h. Trial visits were separated by 3–9 days, with the first trial a minimum of 2 days after completing the preliminary measures. Participants completed a diet diary for 24 h before their first trial and were instructed to repeat their food and fluid intake before their second trial. On arrival at the laboratory, participants provided a venous blood sample (pre‐supplementation) before consuming a capsule containing 3 mg·kg^−1^ body mass of caffeine during the caffeine trial, and an identically looking capsule containing 3 mg·kg^−1^ body mass of microcrystalline cellulose during the placebo trial, with the order of trials randomized. The capsule was swallowed with water (3 mL·kg^−1^ body mass). During a subsequent 30 min of seated rest, participants consumed water *ab libitum* in their first trial, with the same volume consumed during their second trial. Participants then provided a second venous blood sample (pre‐exercise). Forty‐five minutes after consuming the caffeine or placebo capsule, participants commenced 20 min of cycling at a power output corresponding to 70% V̇O_2max_ as determined in the preliminary trial. After 5 min rest, during which participants consumed water (2 mL·kg^−1^ body mass), participants completed a 15 min cycling time trial (during which they were required to complete as much work as possible). Participants then provided a third and final venous blood sample 30 min after the time trial (post‐exercise). Exercise was undertaken in an environmental chamber (temperature 18.4 ± 1.0°C; relative humidity 49.0 ± 7.5%). Participants reported gastrointestinal symptoms (nausea, stomach upset) by questionnaire at the end of each trial visit.

### 2.3 Blood collection and handling

Whole blood samples were collected by venepuncture from a prominent vein in the antecubital fossa into K_2_EDTA vacutainers (Becton Dickinson, Oxford, UK). Blood was centrifuged immediately at 1500*g* for 10 min at 4°C, with plasma frozen at −80°C for later analysis. An aliquot of whole blood was separated for DNA extraction, before being disposed of. Blood was also collected into serum vacutainers for analysis of serum caffeine and paraxanthine, as reported previously (Carswell et al., 2020).

### 2.4 Intestinal fatty acid binding protein

Plasma intestinal fatty acid binding protein (iFABP), as a marker of enterocyte damage, was determined by enzyme‐linked immunosorbent assay (DY3078, Bio‐Techne, Minneapolis, USA), with the procedures optimized for plasma as described previously (Ogden et al., 2020), including supplementing reagent diluents with normal goat serum. All samples for any individual participant were assayed on the same plate. In total, 3 separate assay plates were used, with the intraassay coefficient of variation, calculated per plate, ranging from 2.9% to 5.0%.

### 2.5 ADORA2A and CYP1A2 genotype

Genomic DNA was isolated from whole blood using the Zymo Miniprep Kit (Quick‐DNA Miniprep Plus Kit, Zymo Research, Irvine, California, USA) and immediately frozen at −80°C for analysis after participants had completed the experimental trials. Single‐nucleotide polymorphisms in ADORA2A (rs5751876) and CYP1A2 (rs762551) genes were determined using rhAmp assays (Integrated DNA Technologies, Coralville, Iowa, USA). Participants were categorized by the ADORA2A gene as high sensitivity (TT homozygous) or low sensitivity (C allele carriers: CT heterozygous and CC homozygous) and by the CYP1A2 gene as fast metabolisers (AA) or slow metabolisers (C allele carriers: AC or CC) (see Table 1).

### 2.6 Statistical analysis

These were secondary and exploratory analyses so no a priori sample size calculation was conducted. A sample size estimation was calculated for the primary outcome of endurance exercise performance (Carswell et al., 2020). Four‐way mixed ANOVAs (repeated for treatment [i.e., caffeine vs. placebo] and time, between for each genotype) were used to analyze the temporal response of plasma iFABP between conditions and genotypes. All data were analyzed using IBM SPSS statistics v29 (IBM Corp, Armonk, NY, USA). Statistical significance was accepted at *p* < 0.05.

## 3 RESULTS

**FIGURE 1:**
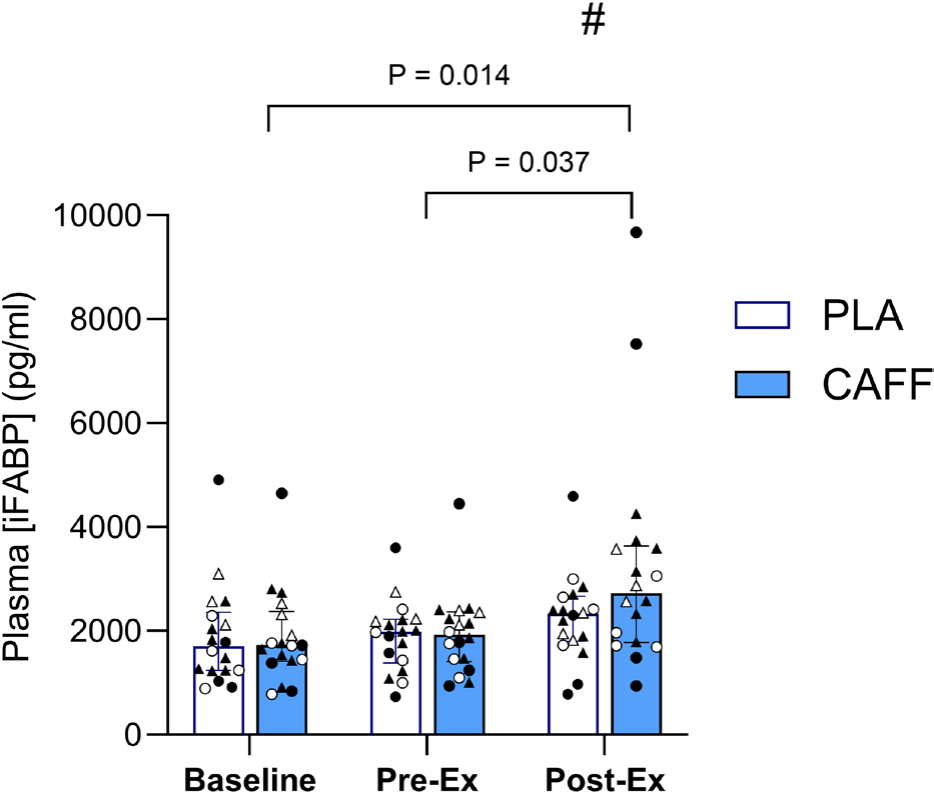
Plasma iFABP for caffeine and placebo trials measured pre‐supplementation, pre‐ and post‐exercise. Bars show median and interquartile range. P values show post hoc follow‐up for time effect. # indicates difference (*p* = 0.010) between CAFF versus PLA for Post‐Ex increase. ADORA TT *n* = 11 (black markers), CT/CC *n* = 7 (white markers); CYP1A2 AA *n* = 10 (triangle markers), AC/CC *n* = 8 (circle markers). Classifications: ADORA2A, TT, high sensitivity; CT or CC: low sensitivity; CYP1A2, AA: fast metaboliser; AC or CC, slow metaboliser.

Plasma iFABP results required log transformation prior to analysis to meet the normal distribution assumption. The four‐way mixed ANOVA (repeated for time and condition, and between‐groups for each of the SNPs) revealed significant main effects of treatment (*p* = 0.031), time (*p* = 0.005 _[Greenhouse‐Geisser corrected]_), and treatment × time interaction (*p* = 0.001), showing an influence of caffeine (Figure 1). Post hoc paired *t*‐tests (Bonferroni corrected) revealed a significant increase following the time trial compared to baseline (*p* = 0.014) and Pre‐Ex (*p* = 0.037), which was greater in the caffeine trial (Figure 1).

**FIGURE 2:**
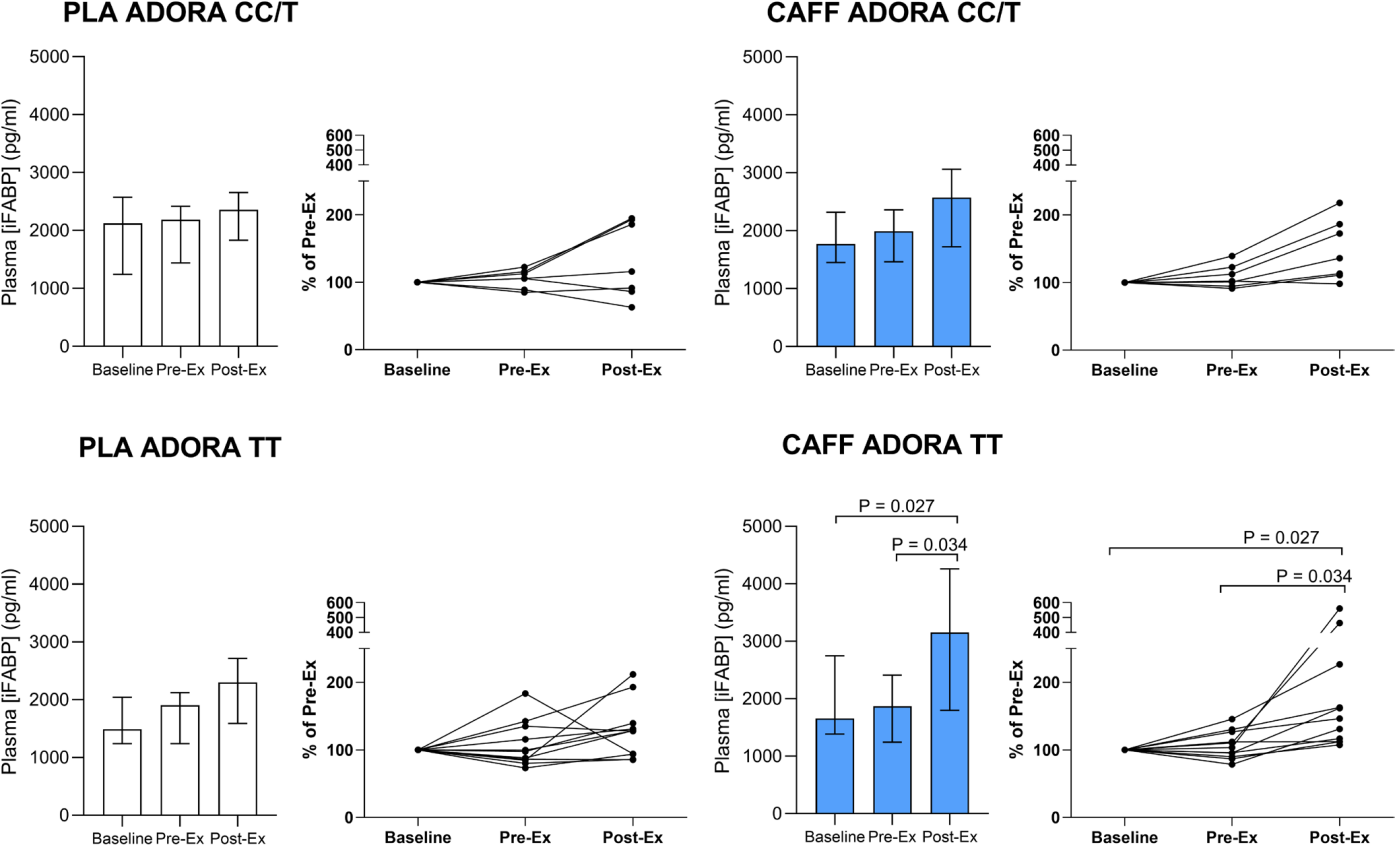
Plasma iFABP for caffeine and placebo trials measured pre‐supplementation, and pre‐ and post‐exercise, with participants categorized by ADORA2A genotype (TT, high sensitivity; CT/CC, low sensitivity). Left panels show PLA trial; Right panels show CAFF trial; Top panels show low sensitivity group; Bottom panels show high sensitivity group. Bars show median and interquartile range. *p* values show post hoc follow‐up for time effect. To follow‐up the interactions involving ADORA2A genotype, two‐way (trial × time) ANOVAs were performed on each ADORA genotype separately: For the CC/CT group there were no main effects of treatment (*p* = 0.779), time (*p* = 0.061 [Greenhouse–Geisser corrected]), or treatment × time interaction (*p* = 0.337 [Greenhouse–Geisser corrected]). For the TT group there were significant main effects of treatment (*p* = 0.003), time (*p* = 0.011 [Greenhouse–Geisser corrected]), and treatment × time interaction (*p* = 0.011), with further post hoc analysis revealing no time effect in the PLA trial (*p* = 0.102), and significant post‐time‐trial increase in the caffeine trial (*p* = 0.027 vs. baseline; *p* = 0.034 vs. Pre‐Ex).

**FIGURE 3:**
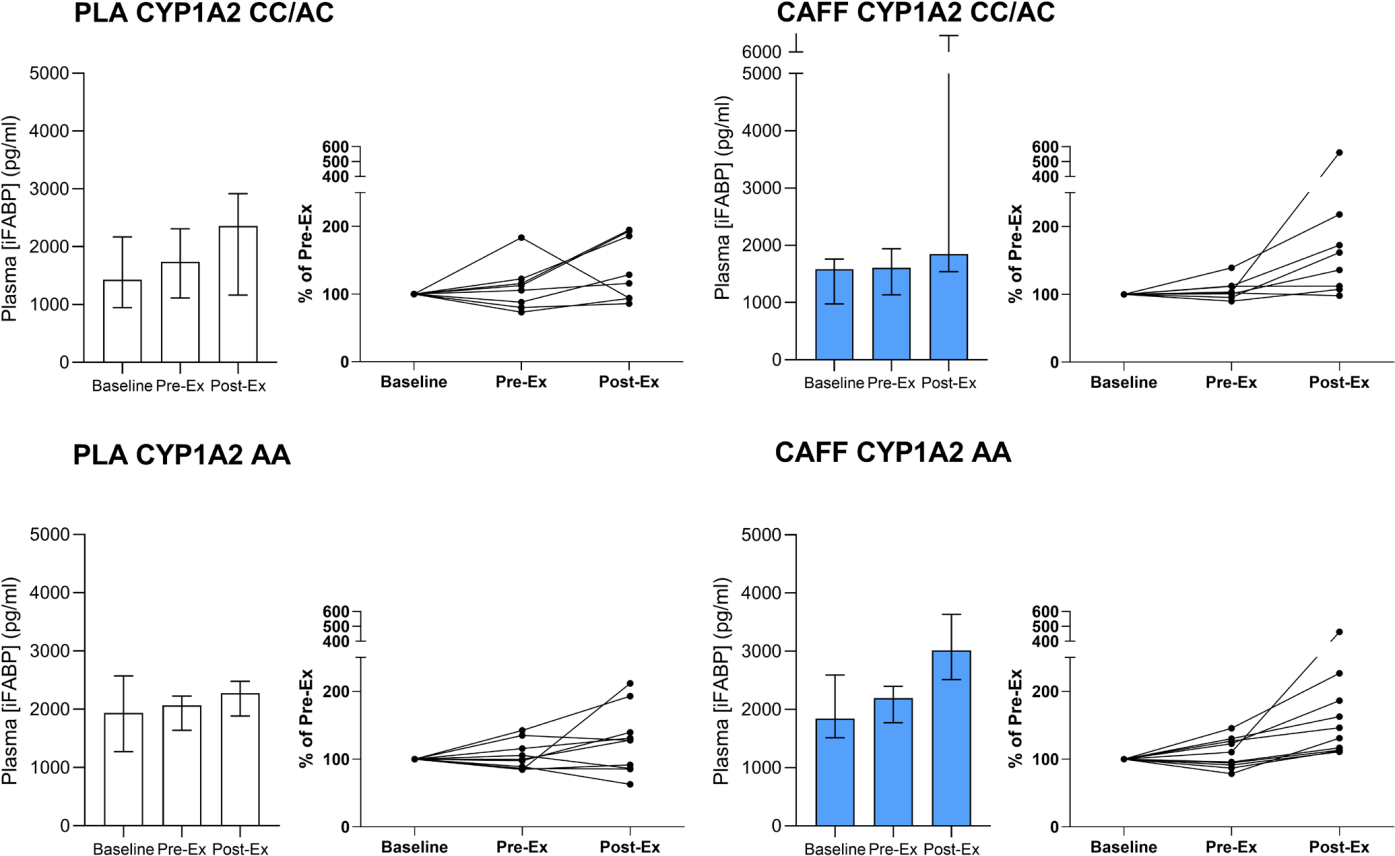
Plasma iFABP for caffeine and placebo trials measured pre‐supplementation, and pre‐ and post‐exercise, with participants categorized by CYP1A2 genotype (AA, fast metaboliser; AC/CC, slow metaboliser). Left panels show PLA trial; right panels show CAFF trial; top panels show slow metabolisers; bottom panels show fast metabolisers. Bars show median and interquartile range.

There was a trial × ADORA2A genotype interaction (*p* = 0.021, see Figure 2), and a trial × time × CYP1A2 × ADORA2A interaction (*p* = 0.005) but no interaction with trial × CYP1A2 genotype alone (*p* = 0.701, see Figure 3). Post hoc follow‐up revealed post‐exercise increases with caffeine only in the ADORA TT categorized group suggesting the overall increase seen with caffeine versus placebo was largely driven by “high‐sensitivity” TT participants. There were no other significant main effects or interactions (see Supplemental data for full table of effects).

### 3.1 Gastrointestinal symptoms

Symptoms (nausea, stomach upset) were reported in 2 of 18 participants in each trial (PLA and CAFF) (see Table 1). In the PLA trial, the reports were from one slow and one fast metaboliser (CYP1A2 SNP categorisations), and both were low sensitivity (ADORA2A SNP categorisations). In the CAFF trial, the reports were from one slow metaboliser (CYP1A2) and low sensitivity ADORA2A (same participant experiencing symptoms in the PLA trial), and the other was a different participant (with no symptoms in the PLA trial) who was a fast metaboliser (CYP1A2) and high sensitivity (ADORA2A). Due to the low number of reported symptoms, no statistical analysis was performed.

**TABLE 1:** Participant gastrointestinal symptom reporting.

Participant	CYP1A2 genotype rs762551	ADORA2A genotype rs5751876	PLA trial		CAFF trial	
			Nausea	Stomach upset	Nausea	Stomach upset
A	AC	TT	No	No	No	No
B	AC	CC	Yes	No	Yes	No
C	AA	CC	Yes	No	No	No
D	AC	TT	No	No	No	No
E	AA	TT	No	No	No	No
F	AA	CC	No	No	No	No
G	AA	TT	No	No	No	No
H	AC	TT	No	No	No	No
I	AA	TT	No	No	No	No
J	AC	CC	No	No	No	No
K	AA	TT	No	No	Yes	No
L	AA	CT	No	No	No	No
M (female)	AC	CC	No	No	No	No
N (female)	AA	TT	No	No	No	No
O (female)	AC	CC	No	No	No	No
P (female)	AA	TT	No	No	No	No
Q (female)	AA	TT	No	No	No	No
R (female)	CC	TT	No	No	No	No

## 4 DISCUSSION

The findings of this study show that acute damage to gut cells caused by endurance exercise may be exacerbated by caffeine, and that this effect is influenced by participants' ADORA2A genotype. Individuals with the rs5751876 homozygous TT genotype (“high‐sensitivity” individuals) showed larger caffeine‐exercise‐induced increases in plasma iFABP. These results provide preliminary mechanistic evidence for a link between caffeine intake and gastrointestinal symptoms reported in previous studies (e.g., de Souza et al., 2022; Pallarés et al., 2013; Wilson, 2016) and support the suggestion from Wilson (2022) that genetic factors may contribute to variability in exercise‐related gastrointestinal responses to caffeine.

Previous research has demonstrated relationships between gut cell damage markers (e.g., iFABP), gastrointestinal permeability, and clinical outcomes or symptoms (e.g., March et al., 2017; Walter et al., 2021). However, direct associations between various markers are not always apparent and are likely more complex than simple linear relationships. For example, minimal clinical thresholds may exist, and such concepts are not well reflected by simple correlations. Indeed, Gaskell et al. (2020) and Costa et al. (2017, 2020) have suggested that only an increase of 1000 pg/mL or more is of clinical relevance in relation to gastrointestinal symptoms risk, suggesting that only increases of at least this magnitude are indicative of substantially elevated risk for endotoxaemia, and the consequent inflammatory profiles and development of gastrointestinal symptoms. Extending this, in a real‐world setting with marathon running (Walter et al., 2021), pre‐to‐post exercise plasma iFABP increases in excess of 1000 pg/mL were reported in the majority of the runners. However, the post‐exercise values were substantially higher in athletes who collapsed after a marathon compared with those who did not, providing evidence for the potential role and importance of the gut barrier in this pathway. They reported an average pre‐to‐post‐exercise increase in plasma iFABP of ~2‐fold in “control” runners, compared to an increase of over 13‐fold in a sub‐group of 8 runners who collapsed during or after the race (with many of these exceeding the upper limit of the assay: 20,000 pg/mL). The mean post‐exercise increase in the current study was between ~1.5 and 2‐fold post‐exercise, which is similar to many other laboratory‐based studies (e.g., Chantler et al., 2021; March et al., 2017; Van Wijck, 2012; Van Wijck et al., 2011). Notably, only 4 of 18 participants experienced an increase >1000 pg/mL in the PLA trial, and 6 of 18 in the CAFF trial, which aligns with the low prevalence of gastrointestinal symptoms reported by these participants. The idea that only increases in iFABP above a certain level may be of clinical concern is further supported when considering that I‐FABP is found in the top layer of the mature enterocytes and whilst increases in plasma confirm damage to the epithelial cells themselves, this does not necessarily reflect compromised integrity of the tight junctions or the barrier per se (Chantler et al., 2021). Following from this, to determine the full clinical implications, studies need to measure a comprehensive range of biomarkers: in addition to markers indicative of epithelial cell damage (as in the present study), markers of barrier disruption (e.g., gastrointestinal permeability), bacterial translocation, systemic inflammation, and manifest symptoms are required to determine the “global” clinical picture (e.g., Costa et al., 2022). However, the current findings provide preliminary evidence showing the effects of caffeine on this marker, and hence the gastrointestinal cell damage pathways, which may have implications for athlete outcomes, and confirms that further research exploring this is warranted.

The low symptom prevalence in the current study would suggest that the level of perturbations seen here (caused by exercise, caffeine, and/or their combination) was not sufficient to induce notable gastrointestinal symptoms in these participants. Nevertheless, we provide evidence that caffeine may exacerbate exercise‐induced gastrointestinal cell damage, and this may have implications for more prolonged or severe exercise that is known to induce larger changes in damage markers, and other relevant biomarkers, and gastrointestinal symptoms. Further study is required, in these contexts, to determine whether caffeine may exacerbate those other outcomes, and whether certain individuals (e.g., certain genotypes) are more susceptible. It is also worthy of note that higher doses of caffeine are generally associated with increased gastrointestinal symptom outcomes (with the 3 mg/kg in the present study considered a low dose in this context). For example, de Souza et al. (2022) performed a systematic review of caffeine side effects, including 25 studies (421 athletes). When they grouped results by low (≤3 mg/kg BM), moderate (3.1–6 mg/kg), and high (>6 mg/kg) doses, gastrointestinal complaints were generally more common with caffeine than with placebo, and the prevalence tended to increase with dose even in non‐endurance sports. It is possible, therefore, that higher doses would elicit greater perturbations, even in shorter or non‐endurance events. It is also interesting that they report a higher prevalence of gastrointestinal symptoms at 24 h post (72%) compared to during or immediately post‐exercise (32%). Any gastrointestinal symptoms experienced by athletes have the potential to compromise performance (de Oliveira et al., 2014; Hoffman & Fogard, 2011). Moreover, caffeine is often consumed by athletes as an ergogenic aid, or as a part of their habitual “daily routine” (e.g. tea, coffee). Either way, any performance benefits may be offset or negated if negative gastrointestinal symptoms are experienced. Furthermore, if symptoms develop later post‐exercise, as noted in de Souza et al. (2022), they could negatively affect subsequent recovery, dietary intake, nutrient absorption, training, or performance (Chantler et al., 2021; van Wijck et al., 2013).

There are several mechanisms that may explain the effects of caffeine on gastrointestinal responses to exercise. Each of these mechanisms could have independent effects, but it is likely that they occur concurrently, and it may be the combined effects that produce outcomes to a greater extent in “sensitive” individuals. One mechanism is that caffeine can inhibit mucus glycoprotein secretion, which contributes to the protective mucus layer and integrity (Hamada et al., 1997). Caffeine can also induce intestinal fluid secretion, which could increase stress and damage to the epithelial lining (Wald et al., 1976). Finally, caffeine (as an SNS‐stimulant) will augment activation of the sympathetic nervous system, and exercise‐induced redistribution of blood flow (Graham & Spriet, 1995) which could exacerbate exercise‐induced disturbance to gastrointestinal barrier function caused by splanchnic hypoperfusion (Van Wijck et al., 2011). Together, these mechanisms explain how caffeine could lead to an increase in intestinal permeability in general. In addition, it is likely that some of the effects of caffeine are more pronounced in “sensitive” individuals (e.g., SNS activation and catecholamine responses), which would explain the greater response in these individuals. Briefly, caffeine's primary ergogenic mechanism of action is attributed to antagonism of adenosine receptors (especially A2A subtype, encoded by the ADORA2A gene), and it is suggested that SNPs in this gene could influence ergogenic (Pickering & Kiely, 2018) and other responses to caffeine. For instance, variations in the ADORA2A gene are associated with increased anxiogenic, emotional, and psychological stress responses to caffeine, which is plausibly linked to SNS activity (Childs et al., 2008; Domschke et al., 2012; Rogers et al., 2010). However, no research has directly compared the effect of variations in the ADORA2A gene on biochemical markers of SNS activity and other relevant parameters (e.g., stress hormones/catecholamine responses). As such, further research with all of these measured in the same study is needed to fully explore this.

## 5 LIMITATIONS

As a preliminary and exploratory study, we only assessed a single biomarker of gut epithelial cell damage. To determine the full clinical implications, further studies are required with measures of a more comprehensive range of biomarkers (e.g., markers of barrier disruption, bacterial translocation, systemic inflammation, and more detailed symptoms analysis to determine the “global” clinical picture (e.g., Costa et al., 2022)). Although participants recorded symptoms within a general “side‐effects” questionnaire, these were limited to nausea and stomach upset, and were only reported as present or not. The use of validated questionnaires and ratings in which symptom severity can be rated on a numerical scale may provide greater insight. This study was limited to a low dose of caffeine and relatively short exercise duration (<1 h). While this provides preliminary mechanistic insights, and it is possible to hypothesize that larger caffeine doses and/or more prolonged and strenuous exercise (alone or in combination) would result in greater perturbations to the gastrointestinal barrier and higher symptom incidence and severity; this remains to be determined. In addition, whilst physiological and perceptual measurements were taken during the fixed‐intensity exercise (see Carswell et al., 2020), none were recorded during the performance trial to confirm the same level of (maximal) effort was exerted in each trial. Including methods to fully account for all of these variables would improve future research in this area. This study included 6 female participants, but we did not standardize the phase of the menstrual cycle for study visits or collect information on hormonal contraceptive use. While such factors may influence physiological responses to exercise, or other factors related to gastrointestinal function, there is evidence that our marker of interest (iFABP), and how it responds to exercise, is not influenced by menstrual cycle phase (Edwards et al., 2025; Matsuda et al., 2024; Snipe & Costa, 2018). It is possible that age could also affect outcomes, but our study participants were drawn from a reasonably homogeneous age group with limited variability (see subject demographics details above), so age was not included as a covariate. These approaches were chosen to reduce unnecessary model complexity, given the modest sample size and unbalanced distribution of males and females, and avoid compromising the primary research question. However, it would be prudent for future research (with larger sample sizes) to take account of, and standardize, factors like menstrual phase or hormonal contraceptive use in females and/or include age and sex as additional factors or covariates. As we did not do this in the present study, the findings remain exploratory and hypothesis generating, but our results demonstrate an influence of caffeine, which may be mediated by the ADORA rs5751876 SNP.

## 6 CONCLUSION

Acute damage to gut epithelial cells caused by endurance exercise may be exacerbated by caffeine. This may be influenced by genetic polymorphisms in the ADORA2A gene, with individuals classified as sensitive (rs5751876 TT homozygous) experiencing a greater increase in a biomarker of gut epithelial cell damage. The potential implications of this for gut/gastrointestinal responses to exercise require further examination.

## FUNDING INFORMATION

No external funding was received.

## CONFLICT OF INTEREST STATEMENT

All authors declare no conflicts of interest.

## Supporting information


Data S1.

## Data Availability

Data are available upon reasonable request.
