# Supplementary material for: Caffeine exacerbates exercise‐induced gut cell damage and is influenced by ADORA2A genotype but not CYP1A2 genotype: A preliminary study
Source: Physiol Rep. 2025 Nov 19;13(22):e70673. doi: 10.14814/phy2.70673 (PMC12631023; doi:10.14814/phy2.70673)
Supplement: Supplementary file 1 — Data S1. [file PHY2-13-e70673-s001.docx]

**Caffeine exacerbates exercise-induced gut cell damage and is influenced by ADORA2A** 1 **genotype but not CYP1A2 genotype: a preliminary study.**

**Supplementary results:**

| **Tests of Within-Subjects Effects and interactions from 4-way mixed ANOVA** | | |
| --- | --- | --- |
|  |  | **P value** |
| ***Trial*** | ***Sphericity Assumed / N/A*** | ***0.031**** |
| Trial x CYP | Sphericity Assumed / N/A | 0.701 |
| ***Trial x ADORA*** | ***Sphericity Assumed / N/A*** | ***0.021**** |
| Trial x CYP x ADORA | Sphericity Assumed | 0.121 |
| ***Time*** | ***Greenhouse-Geisser corrected*** | ***0.005**** |
| Time x CYP | Sphericity Assumed / N/A | 0.619 |
| Time x ADORA | Sphericity Assumed / N/A | 0.637 |
| Time x CYP x ADORA | Sphericity Assumed / N/A | 0.197 |
| ***Trial x Time*** | ***Sphericity Assumed / N/A*** | ***0.001**** |
| Trial x Time x CYP | Sphericity Assumed / N/A | 0.767 |
| Trial x Time x ADORA | Sphericity Assumed / N/A | 0.289 |
| ***Trial x Time x CYP x ADORA*** | ***Sphericity Assumed / N/A*** | ***0.005**** |

Plasma iFABP results required log transformation prior to analysis to meet the normal distribution assumption. The four-way mixed ANOVA (repeated for time, condition, and between groups for each of the SNPs) revealed significant main effects of treatment (P = 0.031), time (P = 0.005 _[Greenhouse-Geisser corrected]_), and treatment × time interaction (P = 0.001), showing an influence of caffeine (Figure 1). Post hoc paired t-tests (Bonferroni corrected) revealed a significant increase following the time-trial compared to baseline (P = 0.014) and Pre-Ex (P = 0.037), which was greater in the caffeine trial (Figure 1).

There was a trial x ADORA2A genotype interaction (P = 0.021, see Figure 2), and trial x time x CYP1A2 x ADORA2A interaction (P = 0.005) but there were no other significant main effects or interactions, including no CYP1A2-specific interactions (trial x CYP1A2 P = 0.701; trial x CYP1A2 x ADORA2A P = 0.121; time x CYP1A2 P = 0.619; time x ADORA2A P = 0.637; time x CYP1A2 x ADORA2A P = 0.197; trial x time x CYP1A2 P = 0.767; trial x time x ADORA2A P = 0.289) showing that the overall effect of trial was influenced by the ADORA2A genotype. To follow-up the interactions involving ADORA2A genotype, 2-way (trial x time) ANOVAs were performed on each ADORA genotype separately: for the CC/CT group there was no main effects of treatment (P = 0.779), time (P = 0.061 _[Greenhouse-Geisser corrected]_), or treatment × time interaction (P = 0.337 _[Greenhouse-Geisser corrected]_). For the TT group there were significant main effects of treatment (P = 0.003), time (P = 0.011 _[Greenhouse-Geisser corrected]_), and treatment × time interaction (P = 0.011), with further post hoc analysis revealing no time effect in the PLA trial (P = 0.102), and significant post-time-trial increase in the caffeine trial (P = 0.027 vs baseline; P = 0.034 vs Pre-Ex), suggesting the overall increase seen with caffeine vs placebo was largely driven by ‘high sensitivity’ participants (see Figures 1-3).
